# Influence of Diet on the Composition of Bone

**Published:** 1871-03

**Authors:** 


INFLUENCE OF DIET ON THE COMPOSITION OF BONE.—M Papillon has communicated to the French Academy some experiments made on pigeons and rats. These animals were fed daily for months, with food containing small quantities of the phosphates of strontia, of magnesia, and alumina. No visible effect was produced in their health. On analysis of the bones these substances were found in them. If these substances can be made to enter into the composition of bones by an appropriate diet, there seem some grounds for hoping that we may be able to modify animal tissues by means of medicine, and this is a subject well worthy of further experimentation.

